# A Bidimensional Model of Language Transmission in Bilingual Families: Immigrants from the Former Soviet Union in Israel

**DOI:** 10.3390/bs16050712

**Published:** 2026-05-06

**Authors:** Eugene Tartakovsky

**Affiliations:** The Bob Shapell School of Social Work, Tel-Aviv University, P.O. Box 39040, Ramat Aviv, Tel Aviv 69978, Israel; evgenyt@tauex.tau.ac.il

**Keywords:** bidimensional acculturation model, dominant language, Hebrew, heritage language, language proficiency, language transmission, Russian

## Abstract

This study investigates language transmission in immigrant families. The study is based on a bidimensional acculturation model, which assumes that immigrants acquire the new culture and preserve their culture of origin to different degrees. The model was tested using a stratified sample of first-generation immigrants from the former Soviet Union in Israel whose children were born in the host country (n = 725). The assimilation pattern was observed across all components of language transmission, with Hebrew being more prevalent than Russian among parents and children, as well as in their interactions. In addition, the two languages were competitive (negatively correlated) with respect to parents’ language proficiency and parent–child interactions. However, they were complementary (non-correlated) with respect to children’s language proficiency. The hypothesized bidimensional model linking parents’ language proficiency, the frequency of parent–child interactions in a specific language, and children’s language proficiency was corroborated for both languages. In addition, positive effects of parents’ proficiency in Russian on children’s proficiency in both Russian and Hebrew were found. Finally, the duration of residence in Israel, religiosity, education, and gender affected various aspects of language transmission in immigrant families. The study’s results advance our understanding of immigrants’ language acculturation and chart new directions for language policy and practice.

## 1. Introduction

Numerous studies have demonstrated the importance of both heritage language (HL), the language of the country of origin, and dominant language (DL), the language of the majority in the host country, for immigrants’ socio-cultural and psychological adjustment and development. Among adult immigrants, DL proficiency was associated with greater social support from the majority society, lower levels of discrimination, and higher psychological well-being, whereas HL was associated with preserving immigrants’ original cultures and social support within the immigrant community (Alshihry, 2024; Tartakovsky, 2025a). Among children of immigrants, higher proficiency in DL was associated with school success, years of post-secondary education, and higher income (Alshihry, 2024; Chiswick & Miller, 2007; Montrul, 2023; Stavans & Ashkenazi, 2022; Sullivan et al., 2021; Troesch et al., 2021). Preserving HL was associated with more effective communication with parents, more parental control and less neglect, reduced parent–child conflict, more secure attachment, better relationships with parents and grandparents, and decreased adolescent vulnerability to deviant peer influences (Cox et al., 2021; DeSouza et al., 2023; Dorner et al., 2023; Kao & Tienda, 2022; Medvedeva, 2012; Otwinowska et al., 2021).

Several studies have investigated language transmission in immigrant families. However, most researchers have assumed that family plays an important role only in HL transmission, while host society institutions (schools, workplaces, and mass media) play a more important role in DL transmission because immigrant parents are more proficient in HL and less proficient in DL. Therefore, most studies have focused on the family role in preserving HL (Bilgory-Fazakas & Armon-Lotem, 2025; Bose et al., 2023; Chiswick & Miller, 2007; Hollebeke et al., 2023; Soehl, 2016), whereas only a few have investigated the transmission of DL in immigrant families (Bezcioglu-Goktolga & Yagmur, 2022; Sullivan et al., 2021; Troesch et al., 2021). Finally, only a few studies have investigated the connection between the two languages during language transmission (Tartakovsky, 2025b).

The present study investigated language transmission in immigrant families by applying a bidimensional model (Berry, 2017; Tatarko & Berry, 2024). Specifically, we assumed that immigrant parents are proficient, albeit to different degrees, in both HL and DL, and that they transmit these languages to their children by interacting with them in both languages (again, to different degrees). Furthermore, we investigated which language prevails among parents and children, and in their interactions, and how the two languages relate to each other.

The study was conducted among first-generation immigrants from the former Soviet Union (FSU) in Israel who had children born in Israel. The main bulk of immigrants from the FSU arrived in Israel more than 30 years ago. Most of them did not know Hebrew upon arrival because studying it was forbidden in the FSU (Leshem & Sicron, 1999; Tartakovsky, 2010, 2025b); however, they have learned the language while living in Israel. On the other hand, the presence of a large Russian-speaking community (about one million, or 10% of the Israeli population) has enabled immigrants from the FSU to preserve their heritage language (Tartakovsky, 2023, 2025b).

### 1.1. The Bidimensional Model

The bidimensional model claims that immigrants answer two fundamental questions: to what degree do they want to preserve their heritage culture, and to what degree do they want to acquire the dominant culture of the host country (Berry, 2017; Tatarko & Berry, 2024). Furthermore, the model assumes the existence of four acculturation strategies: integration (i.e., keeping one’s original culture while also adopting the majority culture), assimilation (i.e., adopting the majority culture and relinquishing one’s heritage culture), separation (i.e., maintaining one’s heritage culture and rejecting the majority culture), and marginalization (i.e., rejecting both the heritage and the majority culture). The two cultural dimensions have been operationalized in various ways, including identification with the two cultures, adherence to their cultural practices, contact with members of the two groups, and mastery of the two languages (Berry et al., 2022; Bierwiaczonek et al., 2023; Francot et al., 2024).

The bidimensional acculturation model postulates that the two cultural dimensions are independent, i.e., immigrants can maintain their native culture and adopt the majority culture to various degrees. However, previous studies have demonstrated the variability of the relationships between them, finding the correlations between the two dimensions to be negative (a concurrent pattern), non-significant (a complementary pattern), or positive (a cumulative pattern) in different groups (Berry et al., 2006; Bierwiaczonek et al., 2023; Safdar et al., 2013; Staerklé et al., 2010; Tartakovsky, 2023). The acculturation strategies were linked to group relations, being positive or neutral when groups have congenial relations and negative in situations of group conflict (Tartakovsky, 2025a, 2025b).

The present study focuses on the heritage (Russian) and dominant (Hebrew) languages among immigrant adults and their children. Several studies have investigated language acculturation among FSU immigrants in Israel (Horenczyk, 2010; Remennick, 2004; Remennick & Prashizky, 2024; Tartakovsky, 2025b). Earlier studies have revealed a separation pattern in language acculturation, with immigrants tending to prefer Russian over Hebrew in everyday use. However, those studies have also found that language preferences changed with time, with Hebrew strengthening and Russian weakening as immigrants live longer in Israel (Tartakovsky, 2010, 2023, 2025b). Recent studies have found an assimilative pattern of language acculturation among FSU immigrants in Israel, with Hebrew prevailing over Russian in everyday use (Tartakovsky, 2025b). Those studies have also found a concurrent pattern of relationships between DL and HL, i.e., a negative correlation in the frequency of their daily use (Tartakovsky, 2025b).

### 1.2. Factors Affecting DL and HL Proficiency in Immigrant Children

#### 1.2.1. Parents’ Language Proficiency

Previous studies have found that parents’ proficiency in HL is connected to children’s proficiency in that language (Cox et al., 2021; Montrul, 2023; Otwinowska et al., 2021; Remennick & Prashizky, 2024; Soehl, 2016; Troesch et al., 2021). Researchers assumed that the main mechanism linking the parents’ and children’s language proficiency lies in parent–child interactions, when parents proficient in HL use this language for communication with their children: they speak with their children and read them books in this language, which contributes to the children’s language development (Dixon & Wu, 2014; Dorner et al., 2023; Medvedeva, 2012; Montrul, 2023). Other joint language-bound activities (e.g., watching TV) have also been assumed to influence language transition in immigrant families (Dixon & Wu, 2014).

The effect of parents’ DL proficiency on children’s proficiency in that language has been rarely investigated, and the results have been inconsistent. Some studies have demonstrated that parents’ DL proficiency affects children’s proficiency in that language; however, other studies have not found this connection (Cox et al., 2021; Dixon & Wu, 2014; Slawny et al., 2025). The effect of parents’ proficiency in DL was attributed to their use of that language for communication with their children. However, the researchers argued that because of parents’ lower proficiency in DL, they might be less able to use that language in parent–child communication. Therefore, the parents’ proficiency in DL had a weaker effect on children’s proficiency in that language (Carmel, 2022; Chavez et al., 2023; Slavkov, 2017).

An important question in immigrant language studies concerns the crisscross effects of parents’ language use: parents’ DL on children’s HL and parents’ HL on children’s DL. Some researchers have assumed that parents’ use of HL at home may negatively affect their children’s DL development. However, others have assumed that parents’ HL use may positively affect children’s DL development by promoting general cognitive development, fostering high expectations for language studies and literacy, and enhancing children’s general literacy skills (Cox et al., 2021; Dixon & Wu, 2014; Carmel, 2022). The results of empirical studies on the crisscross effects of parents’ languages have been inconclusive. Studies in the US have found that among Hispanic immigrant families using English at home has had a positive impact on children’s English skills, but a negative impact on their HL skills, while similar studies among Chinese immigrants found a positive effect of HL use by parents on the proficiency of their children in this language but no effect of parents’ HL on children’s DL (DeSouza et al., 2023; Otwinowska et al., 2021). The direction and size of crisscross connections between parents’ and children’s languages have also varied across immigrant groups in European and Israeli studies (Dixon & Wu, 2014; DeSouza et al., 2023).

#### 1.2.2. Socio-Demographic Factors

Among socio-demographic factors, the effect of residence duration in the host country on DL proficiency was the strongest (Troesch et al., 2021). In addition, there was a strong decline in the use of HL and an increase in the use of DL across the first, second, and third generations in immigrant families (DeSouza et al., 2023). Researchers explained these changes by increased exposure to DL and decreased exposure to HL, the host society’s social pressure to use DL, and the differential utility of the two languages for social adjustment and the psychological well-being of immigrants (DeSouza et al., 2023; Stavans & Ashkenazi, 2022; Tartakovsky, 2010, 2023, 2025a).

Higher education and socio-economic status were associated with higher DL proficiency among adult immigrants and their children (Dixon & Wu, 2014; Dorner et al., 2023; Stavans & Ashkenazi, 2022; Sullivan et al., 2021; Troesch et al., 2021). Well-educated immigrants may have higher general cognitive abilities and language skills, as well as greater motivation to acquire a new language. In addition, more educated parents tend to engage in more literacy activities with their children at home and adapt more easily to mainstream literacy instructions (Dixon & Wu, 2014; Dorner et al., 2023; Sullivan et al., 2021). However, most researchers considered extra-family factors more important for immigrant children’s DL proficiency (Dorner et al., 2023; Troesch et al., 2021).

The role of parents’ education and SES on the adults’ and children’s HL proficiency has been less studied. Researchers assumed that the same factors (e.g., higher cognitive abilities and language skills) may explain the effect of education and SES on HL proficiency among immigrants (Alshihry, 2024; Bilgory-Fazakas & Armon-Lotem, 2025; Chiswick & Miller, 2007). However, some researchers noted a paradox: well-educated parents with a high level of HL proficiency showed little motivation to transmit that language to their children because they considered it of little value for children’s social adjustment in the host country (Bezcioglu-Goktolga & Yagmur, 2022; Soehl, 2016).

Few gender differences in DL and HL proficiency have been found among immigrants in the US (Chiswick & Miller, 2007; Kao & Tienda, 2022; Kaveh & Lenz, 2024). This finding is surprising, given that women, on average, have higher verbal abilities than men (Petersen, 2018). Different exposure to HL and DL and different educational levels among immigrant men and women in some immigrant groups may explain the absence of gender differences in language proficiency, whereby immigrant women from less developed countries are less educated and less exposed to the job market than men (Bezcioglu-Goktolga & Yagmur, 2022; Chiswick & Miller, 2007; Kao & Tienda, 2022; Kaveh & Lenz, 2024; Stavans & Ashkenazi, 2022). However, gender differences in language acculturation among highly educated immigrant groups have not been sufficiently studied.

Another understudied factor in immigrants’ language proficiency is religiosity (Nazari, 2024; Temple et al., 2022). Religious similarity to the majority population may prompt immigrants to work harder to learn DL and become more proficient in it. On the other hand, a higher level of religiosity among immigrants whose religion differs from the host country’s dominant religion may motivate them to preserve HL (Jasemi & Gottardo, 2023). We test these assumptions in the present study.

### 1.3. The Present Study

In this study, we addressed the following research questions: (1) Which language prevails among parents and children, and in their interaction, and how do the two languages relate to each other? (2) How do parents’ proficiency in HL and DL affect their interactions with children and children’s language proficiency? (3) How do socio-demographic factors affect parents’ and children’s language proficiency?

To answer these questions, we formulated and tested a bidimensional model of language transition in immigrant families. We assumed an assimilation–concurrent pattern of language acculturation in families of FSU immigrants in Israel. We further assumed that parents’ language proficiency affects children’s language proficiency through parent–child interactions in both DL and HL. Finally, we assumed that socio-demographic factors affect children’s language proficiency both directly and indirectly, by influencing parents’ language proficiency and parent–child interactions. We formulated the following hypotheses:

1. For each element of language transition (parents’ proficiency, the frequency of interactions, and children’s proficiency), DL prevails over HL, indicating the assimilation language acculturation pattern.
2. Each element of language transition in one language (parents’ proficiency, the frequency of interactions, and children’s proficiency) competes with the corresponding element in the other language (i.e., the correlations between the corresponding elements are negative).
3. For both DL and HL, parents’ language proficiency is connected to the frequency of parent–child interactions in this language, which, in turn, is connected to children’s language proficiency. Thus, parent–child interactions mediate the connection between parents’ and children’s language proficiency in both languages.
4. Socio-demographic variables affect children’s proficiency in both languages directly and indirectly, through their effect on parents’ language proficiency and parent–child interactions.
  (a) Time of residence in Israel, nationality (Jewish vs. other), and religiosity are associated with higher levels of DL and lower levels of HL in all elements of language transmission.
  (b) Parents’ education is associated with higher levels of both DL and HL in immigrant parents and their children.
  (c) Origin from Slavic republics in the FSU (Russia, Belarus, and Ukraine), where Russian is widespread, is associated with higher levels of Russian among immigrant parents and their children.
  (d) Gender is not associated with proficiency in either language.

## 2. Methods

### 2.1. Sampling

The study used a stratified sample of immigrants from the former Soviet Union in Israel who have a child born in Israel (n = 725). Table 1 presents the socio-demographic characteristics of the sample. The sample consisted of 66% females, 65% had a tertiary education, 94% were Jewish, and 71% had emigrated from the three Slavic republics of the FSU. The average age was 44.8 years (*SD* = 9.11), the average time lived in Israel was 33.9 years (*SD* = 8.7), and the average age at arrival in Israel was 10.5 years (*SD* = 6.5). The average age of the children was 15.2 years (*SD* = 10.7), with 12% below the kindergarten age, 9% in kindergarten, and 50% in school.

### 2.2. Procedure

Two survey companies (iPanel and Panel4All) recruited the participants from their panels. All panel members born in the FSU and having a child born in Israel received an invitation to participate in the study, along with a link to the Qualtrics questionnaire. Those who agreed to participate completed the questionnaire and received a standard compensation of approximately $5. Participants could choose a questionnaire in Russian or Hebrew, and 88% chose Hebrew. Participation in the survey was voluntary and anonymous. All participants provided written consent. The sample was stratified by age, gender, and education. The Ethical Review Board at Tel Aviv University approved the study. The study was conducted in November 2025.

### 2.3. Instruments

**Language proficiency.** Following previous studies (Bose et al., 2023), we measured language proficiency in four areas: speech comprehension, talking, reading, and writing. We asked parents to assess their own and their children’s language proficiency in these four areas on a 6-point scale, from 1—*not at all* to 6—*full*. When the participants had several children, they were asked to respond considering their first child born in Israel. Internal consistency of the language proficiency scales was high (Russian/Hebrew) for both parents (Cronbach’s *α* = 0.91/0.95) and children (Cronbach’s *α* = 0.92/0.89).

**Parent–child language interactions.** We measured the frequency of parent–child language interactions in three areas: talking, reading, and watching movies together using a specific language. We asked parents to assess how often they conducted the three activities with their children on a 6-point scale from 1—*never* to 6—*all the time*. Again, when the participants had several children, they were asked to respond considering their first child born in Israel. Internal consistency of the scale was high for both Russian (Cronbach’s *α* = 0.88) and Hebrew (Cronbach’s *α* = 0.82).

**Socio-demographic variables.** The participants reported on the following socio-demographic variables: nationality according to the Israeli Interior Ministry registration (1—*Jewish*, 0—*other*), gender (1—*women*, 2—*men*), time in Israel (*years*), religiosity (from 1—*atheist* to 5—*orthodox*), education (from 1—*no matriculation certificate* to 7—*Ph.D.*), and the republic of origin in the FSU (1—*Slavic republics of Russia, Belarus, and Ukraine*, 0—*all others*).

## 3. Results

### 3.1. Bivariate Analyses

Bivariate analyses were conducted using IBM SPSS Statistics (Version 31). Table 2 presents the means, standard deviations, and correlations for the study variables. To test the assimilation hypothesis (H_1_), i.e., the hypothesis regarding the language prevalence in parents’ language proficiency, parent–child interactions, and children’s language proficiency, we calculated paired-samples *t*-tests. The tests’ results indicated that immigrant parents were more proficient in Hebrew than in Russian (*t*(724) = 17.9, *p* < 0.001, Cohen’s *d* = 1.00) and used more Hebrew than Russian in parent–child interactions (*t*(724) = 26.2, *p* < 0.001, Cohen’s *d* = 1.38); children were also more proficient in Hebrew than in Russian (*t*(724) = 40.9, *p* < 0.001, Cohen’s *d* = 1.32). These results corroborated the hypothesized assimilation pattern of language acculturation in immigrant families.

To further assess language proficiency in DL and HL, we calculated the proportion of parents and children with full non-proficiency in those languages. We found the following proportions of parents and children who reported that they were “not at all” proficient in Russian: 1.5% of parents and 30% of children could not understand conversation and speak Russian, 9% of parents and 63% of children could not read Russian, and 18% of parents and 68% of children could not write in Russian. Regarding Hebrew, only 0.1% of parents reported that they could not understand conversation, speak, read, or write in Hebrew at all. At the same time, immigrant parents reported that 1.5% of their children could not understand conversation in Hebrew, 2.2% could not speak Hebrew, and 13% could not read and write in Hebrew.

To test the languages’ concurrence hypothesis (H_2_), i.e., the hypothesis regarding the relationship between the two languages in immigrant families, we calculated Pearson correlation coefficients. The correlations between the two languages were negative for parents’ language proficiency (*r* = −0.19, *p* < 0.001) and parent–child interactions (*r* = −0.44, *p* < 0.001) but were non-significant for children’s language proficiency (*r* = 0.06, *p* = 0.114). These results corroborated the hypothesized concurrent relationships between DL and HL for immigrant parents but not for their Israel-born children.

### 3.2. Multivariable Analyses

To test the hypotheses regarding connections among the variables (H_3_ and H_4_), we used structural equation modeling in Mplus (Version 9). The following language-related variables were included in the model: parents’ proficiency in Hebrew and Russian, frequency of parent–child interactions in Hebrew and Russian, and children’s proficiency in Hebrew and Russian. Additionally, six socio-demographic variables were included in the model and linked to all the other variables: nationality, gender, time of residence in Israel, religiosity, education, and the republic of origin in the FSU. Parents’ age and age at arrival, as well as children’s age, were excluded from the analysis because they were strongly correlated with each other and with time of residence in Israel. Full information maximum likelihood estimation with robust standard errors was used to address missing data (Enders, 2025; Kelloway, 2014; Little & Rubin, 2019). After establishing the model’s goodness-of-fit and aiming for the most parsimonious model, the model was “trimmed,” i.e., all non-significant paths were excluded (Kelloway, 2014; Kline, 2023). The direct and indirect effects were tested using the bootstrapping method with 1000 resamples and a 95% confidence interval.

**Figure 1:**
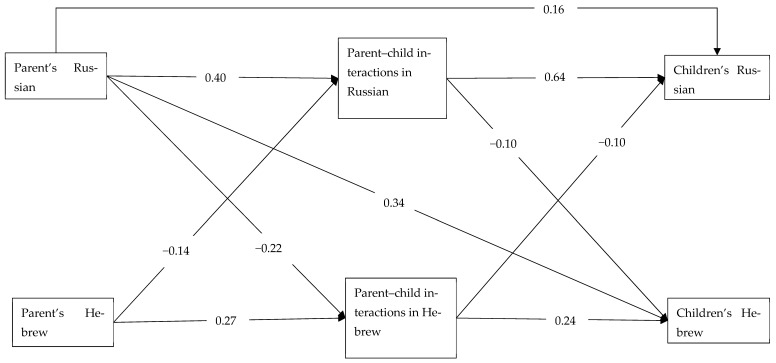
Trimmed model (standardized estimates).

The model demonstrated an excellent fit: *χ*^2^(14) = 15.0, *p* = 0.378; *RMSEA* = 0.010, *CI* (0.000–0.038); *CFI* = 0.999; *TLI* = 0.998; *SRMR* = 0.017. It explained a significant proportion of variance in all variables (*p* < 0.001): parents’ proficiency in Russian (21%), parents’ proficiency in Hebrew (9%), parent–child interactions in Russian (34%), parent–child interaction in Hebrew (20%), children’s proficiency in Russian (62%), and children’s proficiency in Hebrew (21%). Figure 1 presents significant direct connections between variables (standardized estimates). All paths presented in Figure 1 are significant at *p* < 0.05 or a higher level. The figure does not show the effects of socio-demographic variables for better readability.

#### 3.2.1. Effects of Parents’ Language Proficiency and Usage

Confirming hypothesis H_3_, parents’ language proficiency was connected to the frequency of interactions with children, for both Russian (*β* = 0.40) and Hebrew (*β* = 0.27). Further confirming hypothesis H_3_, the frequency of parent–child interactions in each language was connected to the children’s language proficiency for both Russian (*β* = 0.64) and Hebrew (*β* = 0.24). In addition, we found direct positive effects of parents’ proficiency in Russian on children’s proficiency in both Russian (*β* = 0.16) and Hebrew (*β* = 0.34). Finally, we found crisscross negative effects of languages: parents’ proficiency in Russian was negatively connected to parent–child interactions in Hebrew (*β* = −0.22) and parents’ proficiency in Hebrew was negatively connected to parent–child interactions in Russian (*β* = −0.14); parent–child interactions in Russian were negatively connected to children’s proficiency in Hebrew (*β* = −0.10) and parent–child interactions in Hebrew were negatively connected to children’s proficiency in Russian (*β* = −0.10).

To assess the relative strength of Russian and Hebrew in language transmission, we compared their effects using the CONSTRAINT procedure in Mplus. We found that the positive effect of parents’ proficiency in Russian on parent–child interactions in that language was greater than the effect of parents’ proficiency in Hebrew on parent–child interactions in that language (*Estimate* = 0.177, *S.E.* = 0.089, *p* = 0.046). In addition, the positive effect of using Russian in parent–child interactions on children’s proficiency in Russian was greater than the effect of using Hebrew in parent–child interactions on children’s proficiency in Hebrew (*Estimate* = 0.361, *S.E.* = 0.043, *p* = 0.020). The crisscross negative effects did not differ for Hebrew and Russian.

As hypothesized, parents’ proficiency in Russian was indirectly connected to children’s proficiency in Russian (*β* = 0.274, *p* < 0.001) and Hebrew (*β* = −0.092, *p* < 0.001). Total effects of parents’ proficiency in Russian on children’s language proficiency in both languages were positive: in Russian (*β* = 0.438, *p* < 0.001) and Hebrew (*β* = 0.247, *p* < 0.001). In addition, parents’ proficiency in Hebrew was indirectly connected to children’s proficiency in Russian (*β* = −0.115, *p* < 0.001) and Hebrew (*β* = 0.078, *p* < 0.001).

#### 3.2.2. Effects of Socio-Demographic Variables

Time in Israel was directly connected to parents’ proficiency in Hebrew (*β* = 0.24) and Russian (*β* = −0.45), parent–child interactions in Hebrew (*β* = 0.09) and Russian (*β* = −0.23), and children’s proficiency in Hebrew (*β* = 0.34). Religiosity was connected to parents’ proficiency in Russian (*β* = −0.09), parent–child interactions in Hebrew (*β* = 0.08), and children’s proficiency in Hebrew (*β* = 0.11). Gender (1—*women*, 2—*men*) was connected to parents’ proficiency in Russian (*β* = 0.12) and Hebrew (*β* = −0.16) and parent–child interactions in Hebrew (*β* = −0.08). Education was connected only to parents’ proficiency in Hebrew (*β* = 0.12). The FSU republic of origin and nationality (Jews vs. others) were not connected to any variable in the study.

The following socio-demographic variables were indirectly connected to children’s proficiency in Russian: time in Israel (*β* = −0.377, *p* < 0.001), religiosity (*β* = −0.047, *p* = 0.002), education (*β* = −0.014, *p* < 0.001), and gender (*β* = 0.081, *p* < 0.001). The following socio-demographic variables were indirectly connected to children’s proficiency in Hebrew: time in Israel (*β* = 0.047, *p* < 0.001) and education (*β* = 0.009, *p* = 0.003). The following socio-demographic variables were indirectly connected to parent–child interactions in Russian: time in Israel (*β* = −0.210, *p* < 0.001), religiosity (*β* = −0.036, *p* = 0.007), and gender (*β* = 0.017, *p* = 0.007). The following socio-demographic variables were indirectly connected to parent–child interactions in Hebrew: time in Israel (*β* = 0.162, *p* < 0.001), religiosity (*β* = 0.019, *p* = 0.014), and gender (*β* = −0.072, *p* < 0.001).

Summarizing the results on the effects of socio-demographic variables, we may say that hypotheses H_4a_ and H_4b_ regarding the effects of time in Israel, religiosity, and parents’ education were mostly corroborated. At the same time, hypotheses H_4c_ and H_4d_ regarding nationality, republic of origin in the FSU, and gender were not corroborated.

## 4. Discussion

In this study, we investigated language transmission in families of FSU immigrants in Israel, applying a bidimensional acculturation model. We studied language acculturation patterns among immigrant parents and their children, as well as in their interactions. In addition, we investigated the language transmission from immigrant parents to their children through parent–child interactions in both languages. Finally, we examined the effect of socio-demographic variables on language transmission. In what follows, we discuss the results obtained.

### 4.1. Acculturation Patterns in Language Transmission

First, we investigated language preferences among immigrant parents and children, as well as in their interactions. We found that DL prevailed over HL across all aspects of language transmission, indicating an assimilative pattern of language acculturation in FSU immigrant families in Israel. This finding corroborates previous studies on language use among FSU immigrants in Israel (Tartakovsky, 2025b) and broadens our understanding of language acculturation among adults, children, and in parent–child interactions. This finding indicates that the assimilative language acculturation pattern among immigrant children matches that of their parents, and it may be due to the assimilative acculturation pattern prevailing in parent–child interactions (Hoff & Shanks, 2024).

The prevalence of DL over HL was strongest in parent–child interactions and children’s language proficiency and weakest in parents’ language proficiency. These findings indicate that immigrant parents are more bilingual than their children and that the loss of bilingualism in immigrant children may be due to an assimilative pattern of parent–child interactions. Previous studies have noted negative social and psychological consequences of the loss of bilingualism among second-generation immigrants in the US (Bialystok, 2009; Cook, 2014; Troesch et al., 2025). Further studies should investigate its consequences in Israel.

Second, we investigated relationships between the two languages. We found that DL and HL were concurrent (negatively correlated) in parents’ proficiency and parent–child interactions, but were additive (non-correlated) in children’s proficiency. These differences are likely due to differences in language acquisition between immigrant parents and their children. Immigrant parents follow a sequential pattern of language acquisition; i.e., they acquire DL after mastering HL, whereas immigrant children follow a simultaneous pattern; i.e., they acquire the two languages simultaneously (Johnson & Hoff, 2025). In addition, adult immigrants initially acquire DL in educational settings through formal instruction, whereas immigrant children primarily acquire both DL and HL spontaneously through social interactions (Johnson & Hoff, 2025; Klein, 1996; Wibowo et al., 2024). Thus, for parents, the two languages compete for their time and cognitive resources: the more they devote themselves to one language, the less they have for the other. For children, acquiring the two languages requires less cognitive effort; therefore, they compete less with each other. This finding corroborates previous studies of language development among immigrant children (Hoff & Shanks, 2024; Johnson & Hoff, 2025) and advances our understanding of language acquisition and bilingualism.

### 4.2. The Mechanisms of Language Transmission in Immigrant Families

In this study, we corroborated the hypothesis that immigrant parents transfer both DL and HL to their children by interacting with them in those languages. Thus, language-bound parent–child interactions, such as talking, reading, and watching movies together, mediate the connection between parents’ and their children’s proficiency in each language. This finding corroborates previous studies on transmission of HL (Cox et al., 2021; Hoff & Shanks, 2024; Otwinowska et al., 2021; Soehl, 2016; Troesch et al., 2021); however, it advances our knowledge by demonstrating that the language transmission processes in immigrant families are similar for DL and HL.

We further found that parents’ language proficiency had a stronger effect on parent–child interactions and that these interactions, in turn, had a stronger effect on children’s language proficiency in HL than in DL. These findings indicate that immigrant parents have a stronger impact on their children’s HL than on their DL (Hoff & Shanks, 2024). As previous studies have demonstrated, immigrant children are exposed to HL mostly within the family, whereas they are more exposed to DL outside the family (Cox et al., 2021; DeSouza et al., 2023; Hoff & Shanks, 2024). Thus, the different effects of parents in transmitting HL and DL to their children may be explained by different degrees of exposure to these languages at home and in the extrafamilial environment (Johnson & Hoff, 2025).

We found a negative crisscross pattern of language transition for both languages: for parents’ language proficiency on parent–child interactions and for these interactions on children’s language proficiency. It means that the more proficient parents are in one language, the less they use another language in interactions with their children. Furthermore, the more parents use one language when interacting with their children, the less proficient their children become in another language. Thus, the transmission of one language hinders the transmission of the other in immigrant families, probably because one language competes with the other for parents’ and children’s time and cognitive resources within the family. These findings advance our understanding of language transition in immigrant families beyond what has been reported in previous studies (Johnson & Hoff, 2025).

Finally, we found direct positive connections between the parents’ proficiency in HL and children’s proficiency in both HL and DL. Moreover, although the indirect effect of parents’ proficiency in HL on children’s proficiency in DL was negative (because of its negative connection with parent–child interactions in DL and positive connection with parent–child interactions in HL), the total effect of parents’ proficiency in HL on children’s proficiency in DL was positive. Thus, parents’ proficiency in HL positively affected children’s proficiency in both HL and DL. Scholars suggested several psychological mechanisms that might explain these results (Alshihry, 2024; Cox et al., 2021; Dixon & Wu, 2014). First, parents with high HL proficiency may have high standards for language learning. Therefore, they encourage their children to learn both languages (Troesch et al., 2025). Second, parents with high HL proficiency may help their children develop general cognitive and language skills that enable them to master both languages (Montrul, 2023). Finally, parents with high proficiency in HL may provide their children with a more language-rich intra- and extrafamilial environment (e.g., having more books at home and paying for children’s language lessons), which promotes children’s language learning (Hollebeke et al., 2023). Further studies should test the proposed psychological mechanisms and their roles in language transmission in immigrant families.

### 4.3. The Effect of Socio-Demographic Variables

Time of residence in Israel was associated with higher parents’ proficiency and more frequent parent–child interactions in DL and lower proficiency and less frequent interactions in HL. In addition, it was associated with higher proficiency in DL among children. Thus, parents’ and children’s language assimilation increases with time. The increased exposure to DL, social pressure to use DL rather than HL, and the higher utility of DL compared to HL for social adjustment may explain these results (DeSouza et al., 2023; Dixon & Wu, 2014; Hoff & Shanks, 2024; Sullivan et al., 2021; Temple et al., 2022; Troesch et al., 2021).

Another variable connected to language transmission in immigrant families was parents’ religiosity. Its higher levels were associated with parents’ lower HL proficiency, more frequent parent–child interactions in DL, and higher children’s DL proficiency. Previous studies among FSU immigrants in Israel have demonstrated that higher religiosity is associated with stronger Israeli identity and weaker immigrant identity (Tartakovsky, 2023, 2025a). Thereby, higher religiosity may increase parents’ and children’s motivation to master DL and decrease their motivation to preserve HL as a way to strengthen their Israeli identity.

Unlike previous studies, female immigrants from the FSU in Israel reported higher proficiency in DL and more frequent interactions with their children in that language. In their turn, men reported higher proficiency in HL. These results contradict previous findings showing gender similarity in language proficiency (Alshihry, 2024; Hollebeke et al., 2023). The reasons for the gender differences in language transmission in the studied population are unclear, and further research is required to explain them.

Socio-demographic variables affected children’s proficiency in both languages, not only directly but also indirectly through their influence on parents’ language proficiency and parent–child interactions. Parents’ time in Israel and religiosity indirectly positively affected children’s DL proficiency and negatively affected their HL proficiency. In addition, education (negatively) and gender (positively) indirectly affected children’s HL proficiency. Thus, the present study’s results explained the connections between socio-demographic variables and language proficiency in immigrant children reported in previous studies (e.g., DeSouza et al., 2023; Sullivan et al., 2021), demonstrating that these variables affect children’s language proficiency through their influence on parents’ language proficiency and parent–child interactions.

### 4.4. Limitations

This study has several limitations. First, it relied on self-reports, a method prone to reference bias, defined as a systematic error arising from differences in the implicit standards by which individuals evaluate their behavior (Lira et al., 2022). Further studies may use objective measures of language proficiency. Second, in this study, parents reported on their children’s language proficiency, which is problematic because of cultural bias, as parents may systematically overestimate their children’s HL proficiency and underestimate DL proficiency (Runge & Soellner, 2024). In further studies, children’s data may be collected separately. Third, the study sample included one parent from each family. Future studies may adopt a dyadic (parent–child) or triadic (both parents and a child) design, thereby enabling a better understanding of language transmission in immigrant families (Quigley & Nixon, 2022). Fourth, in the present study, we did not control for extrafamilial influences on language development among immigrant children, despite their potential interactions with the language transition within the family (Troesch et al., 2025). Further studies may examine the effects of formal education and the social environment on the language development of immigrant children, as well as their interactions with intrafamilial factors. Finally, the present study was cross-sectional, which did not permit causal conclusions (Savitz & Wellenius, 2023). Further study may employ a longitudinal design to better understand the developmental aspects of language transmission in immigrant families.

### 4.5. Conclusions

The results of the present study advance acculturation theory by corroborating a bidimensional model of language transmission in immigrant families. We have demonstrated that immigrant parents transmit both DL and HL to their children through interaction in those languages. Moreover, we found cross-language negative effects, indicating that the two languages compete for parents’ and children’s time and cognitive resources. However, we have also found that parents’ proficiency in HL promotes children’s proficiency in both HL and DL, likely by strengthening children’s general cognitive and language skills. Furthermore, we have demonstrated that parents’ influence on children’s language proficiency is stronger in HL than in DL. Finally, our findings demonstrate that socio-demographic variables affect children’s language proficiency both directly and indirectly, through their connections with parents’ language proficiency and parent–child interactions.

The results yield several practical recommendations. First, to improve immigrant children’s DL proficiency, the host society should invest more in immigrant parents’ DL proficiency. In addition, immigrant parents should be encouraged to interact more with their children in this language. Second, the host society should prevent the decline in immigrant children’s HL proficiency. It may do so by promoting HL proficiency among immigrant parents and encouraging their interactions with their children in that language. High HL proficiency in immigrant parents is crucial because it is associated with high proficiency in both HL and DL in their children.

## Figures and Tables

**Table 1:** Socio-demographic characteristics of the sample.

Socio-Demographic Characteristics	
Age, M (*SD*)	44.8 (9.11)
Time in Israel, years, M (*SD*)	33.9 (8.7)
Age at arrival to Israel, M (*SD*)	10.5 (6.5)
Age of the child, M (*SD*)	15.2 (10.7)
The religiosity level, M (*SD*)	2.07 (0.67)
Education, M (*SD*)	4.60 (1.32)
Gender, % of females	66%
Nationality, % of Jewish	94%
Origin in the Slavic republics of the USSR	71%

Note: Religiosity (from 1—atheist to 5—orthodox); education (from 1—*no matriculation certificate* to 7—*Ph.D.*).

**Table 2:** Means, standard deviations, and bivariate correlations.

Variables	Parent’s Russian	Parent’s Hebrew	Parent–Child Interactions in Russian	Parent–Child Interactions in Hebrew	Children’s Russian	Children’s Hebrew
Parent’s Russian	1					
Parent’s Hebrew	−0.19 ***	1				
Parent–child interactions in Russian	0.52 ***	−0.27 ***	1			
Parent–child interactions in Hebrew	−0.32 ***	0.34 ***	−0.44 ***	1		
Children’s Russian	0.53 ***	−0.25 ***	0.77 ***	−0.44 ***	1	
Children’s Hebrew	0.05	0.05	−0.18 ***	0.27 ***	0.06	1
*M* (*SD*)	4.71 (1.34)	5.73 (.55)	2.60 (1.49)	4.89 (1.26)	2.44 (1.40)	5.20 (1.25)

Note: *** *p* < 0.001.

## Data Availability

Data may be obtained from the author by requesting.
